# Simultaneous quantitation of chloroquine and primaquine by UPLC-DAD and comparison with a HPLC-DAD method

**DOI:** 10.1186/s12936-015-0570-1

**Published:** 2015-01-28

**Authors:** Tiago A Miranda, Pedro HR Silva, Gerson A Pianetti, Isabela C César

**Affiliations:** Departamento de Produtos Farmacêuticos, Faculdade de Farmácia, Universidade Federal de Minas Gerais, Av Pres Antônio Carlos 6627, 31270-901 Belo Horizonte, MG Brazil

**Keywords:** Chloroquine, Primaquine, Anti-malarials, UPLC-DAD, Tablets

## Abstract

**Background:**

Chloroquine and primaquine are the first-line treatment recommended by World Health Organization for malaria caused by *Plasmodium vivax*. Since the problem of counterfeit or substandard anti-malarials is well established all over the world, the development of rapid and reliable methods for quality control analysis of these drugs is essential. Thus, the aim of this study was to develop and validate a novel UPLC-DAD method for simultaneously quantifying chloroquine and primaquine in tablet formulations.

**Methods:**

The UPLC separation was carried out using a Hypersil C_18_ column (50 × 2.1 mm id; 1.9 μm particle size) and a mobile phase composed of acetonitrile (A) and 0.1% aqueous triethylamine, pH 3.0 adjusted with phosphoric acid (B), at a flow rate 0.6 mL/min. Gradient elution was employed. UV detection was performed at 260 nm. UPLC method was fully validated and the results were compared to a conventional HPLC-DAD method for the analysis of chloroquine and primaquine in tablet formulations.

**Results:**

UPLC method was shown to be linear (*r*^*2*^ > 0.99), precise (CV < 2.0%), accurate (recovery rates from 98.11 to 99.83%), specific, and robust. No significant differences were observed between the chloroquine and primaquine contents obtained by UPLC and HPLC methods. However, UPLC method promoted faster analyses, better chromatographic performance and lower solvent consumption.

**Conclusions:**

The developed UPLC method was shown to be a rapid and suitable technique to quantify chloroquine and primaquine in pharmaceutical preparations and may be successfully employed for quality control analysis.

## Background

Malaria is the world’s most important parasitic infection, ranking among the major health and developmental challenges for the poor countries of the world [1]. Globally, an estimated 3.4 billion people are at risk of malaria. Malaria may be caused by five species of parasites, and *Plasmodium falciparum* and *Plasmodium vivax* are the most important. *Plasmodium falciparum* malaria is the most deadly form, while malaria caused by *P. vivax* has a wider distribution, since it is able to develop in the *Anopheles* mosquito vector at lower temperatures, and to survive at higher altitudes and in cooler climates. It also has a dormant liver stage that enables it to survive during periods when *Anopheles* mosquitoes are not present to continue transmission [1,2].

**Figure 1:**
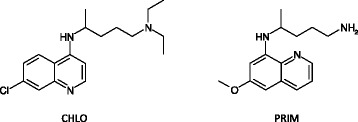
**Chemical structures of chloroquine (CHLO) and primaquine (PRIM).**

The objective of treating malaria caused by *P. vivax* is to cure both the blood stage and the liver stage infections and, thereby, prevent both recrudescence and relapse, respectively. According to the World Health Organization, treatment of *P. vivax* should include an effective schizontocidal medicine, such as chloroquine, combined with a 14-day course of primaquine to prevent relapse (Figure 1) [3,4].

The use of counterfeit and/or substandard anti-malarial drugs can cause increased morbidity and mortality, adverse effects due to excessive dose or the presence of potentially toxic active ingredients or pathogenic contaminants, and the selection of resistant parasites with sub-therapeutic amounts of active ingredient [5]. In addition, treatment failure may be improperly attributed to drug resistance when the product does not meet the qualitative and quantitative requirements [6]. Hence, quality control of anti-malarial pharmaceutical preparations marketed nowadays may help to assure treatment efficacy and avoid development of resistance to anti-malarial drugs [7].

Ultra-performance liquid chromatography (UPLC) is a novel advance in rapid, sensitive and high-resolution liquid chromatography [8]. An ultra-high pressure system allows the use of small particle-packed columns with small diameter. The particles are designed to be able to resist high back pressures, in contrast with conventional liquid chromatography [9]. Therefore, UPLC has significant theoretical advantages in speed, resolution and sensitivity of analysis, especially in time saving and solvent consumption [10,11].

Some papers have described the quantitation of chloroquine or primaquine in pharmaceutical formulations, using liquid chromatography coupled to ultraviolet [12,13] or mass spectrometric detection [14]. Dwivedi *et al.* [15] described the simultaneous quantitation of chloroquine, primaquine and bulaquine by a HPLC method with a long run time of 20 min. However, there are no reported methods regarding the simultaneous quantitation of chloroquine and primaquine using UPLC. Hence, the aim of this study was to develop and validate a novel UPLC-DAD method for simultaneously quantifying chloroquine and primaquine. The validated method was compared to a conventional HPLC-DAD method for the analysis of these anti-malarial drugs in tablet formulations.

## Methods

### Samples and chemicals

Chloroquine diphosphate and primaquine diphosphate reference standards were purchased from Farmacopeia Brasileira (Brasília, DF, Brazil). Chloroquine diphosphate tablets containing 150 mg chloroquine base and primaquine diphosphate tablets containing 15 mg primaquine base were produced by Farmanquinhos/Fundação Oswaldo Cruz (Rio de Janiero, RJ, Brazil). Ultra-pure water was obtained from a Millipore system (Bedford, MA, USA). Acetonitrile and methanol (HPLC grade) were purchased from Tedia (Fairfield, OH, USA) and phosphoric acid and triethylamine (analytical grade) were from JT Baker (Phillipsburg, NJ, USA).

### HPLC analytical conditions

The HPLC analyses were carried out on an Acella system from Thermo Scientific (Waltham, MA, USA), composed of quaternary pump, autosampler and diode array detector (DAD). The column was an ACE C_18_ (100 × 4.6 mm id; 5 μm particle size) from ACT (Aberdeen, Scotland), maintained at 25°C. UV detection was performed at 260 nm and injection volume was 10 μl. The mobile phase was composed of acetonitrile (A) and 0.1% aqueous triethylamine, pH 3.0 adjusted with phosphoric acid (B), at a flow rate 1 mL/min. The gradient elution programme was 10% A from 0–1.9 min, 10-40% A from 1.9-2.0 min and 40% A from 2.0-3.3 min. For column re-equilibration, 10% A was maintained from 3.3-5.00 min.

### UPLC analytical conditions

The analytical conditions employed for the UPLC method were adapted from the HPLC method previously developed and used for routine analyses. The UPLC analyses were carried out on an Acella system from Thermo Scientific (Waltham, MA, USA), composed of quaternary pump, autosampler and DAD. The column was a Hypersil C_18_ (50 × 2.1 mm id; 1.9 μm particle size) from Thermo Scientific (Waltham, MA, USA), maintained at 25°C. UV detection was performed at 260 nm. UV spectra from 200 to 400 nm were on line recorded for peak identification. The injection volume was 7.0 μl. The mobile phase was composed of acetonitrile (A) and 0.1% aqueous triethylamine, pH 3.0 adjusted with phosphoric acid (B), at a flow rate 0.6 mL/min. The separation of chloroquine and primaquine was evaluated in different proportions of these solvents and, for each condition, retention time and resolution (*R*) were calculated. The optimized condition was achieved using a gradient elution programme: 10% A from 0–0.45 min, 10-40% A from 0.45-0.47 min and 40% A from 0.47-1.30 min. For column re-equilibration, 10% A was maintained from 1.30-4.00 min.

### Preparation of solutions

#### Chloroquine and primaquine standard solution

Approximately 20.16 mg of chloroquine diphosphate reference standard (corresponding to 12.50 mg of chloroquine base) were accurately weighed and transferred to a 25-ml volumetric flask. Twenty mL of methanol and 0.1% aqueous triethylamine pH 3.0 (1:1) were added to ensure complete dissolution and solution was diluted to volume using the same solvent. Approximately 21.95 mg of primaquine diphosphate reference standard (corresponding to 12.50 mg of primaquine base) were accurately weighed and transferred to a different 25-ml volumetric flask. Twenty mL of methanol and 0.1% aqueous triethylamine pH 3.0 (1:1) were added to ensure complete dissolution and solution was diluted to volume using the same solvent. Then, aliquots of 3 mL chloroquine stock solution and 2 mL primaquine stock solution were transferred to a 10-ml volumetric flask. This solution was diluted to volume, obtaining final concentrations of 0.15 mg/mL chloroquine and 0.10 mg/mL primaquine.

#### Chloroquine and primaquine sample solution

Chloroquine and primaquine tablets were previously weighed and finely powdered. An accurately weighed portion of chloroquine tablet powder, equivalent to about 12.50 mg chloroquine base, was transferred to a 25-ml volumetric flask followed by the dissolution with methanol and 0.1% aqueous triethylamine pH 3.0 (1:1). In parallel, an accurately weighed portion of primaquine tablet powder, equivalent to about 10.00 mg primaquine base, was transferred to a 50-ml volumetric flask followed by the dissolution with methanol and 0.1% aqueous triethylamine pH 3.0 (1:1). Then, aliquots of 3 mL chloroquine solution and 5 mL primaquine solution were transferred to a 10-ml volumetric flask. This solution was diluted to volume, obtaining final concentrations of 0.15 mg/mL chloroquine and 0.10 mg/mL primaquine.

### Validation

Both HPLC and UPLC methods were fully validated, according to International Conference on Harmonization Guideline - validation of analytical procedures [16]. All the described parameters were evaluated for both methods and the obtained results were compared.

### Linearity

Standard solutions containing 0.30 mg/mL of chloroquine and 0.20 mg/mL of primaquine were prepared, in triplicate. Aliquots of these solutions were diluted in methanol and 0.1% aqueous triethylamine pH 3.0 (1:1) to five different concentrations, corresponding to 0.09, 0.12, 0.15, 0.18, and 0.21 mg/mL of chloroquine and 0.06, 0.08, 0.10, 0.12, and 0.14 mg/mL of primaquine. Linear ranges corresponded from 60% to 140% of test concentration for each drug. Calibration curves for concentration *versus* peak area were plotted for each compound and the obtained data were subjected to regression analysis using the least squares method.

### Precision

The intra-day precision was evaluated by analysing six samples (*n* = 6), at 100% of the test concentration (0.15 mg/mL of chloroquine and 0.10 mg/mL of primaquine). Similarly, the inter-day precision was evaluated in two consecutive days (*n* = 12). Chloroquine and primaquine concentrations were determined and the coefficient of variation (CV%) were calculated.

### Accuracy

Recovery was investigated by means of a standard addition experiment. Different volumes of standard solutions containing 0.30 mg/mL of chloroquine and 0.20 mg/mL of primaquine were added to a sample solution prepared with chloroquine and primaquine tablets. These solutions were further diluted with methanol and 0.1% aqueous triethylamine pH 3.0 (1:1), obtaining three levels: 75, 100 and 125% of the label claim. At each level, samples were prepared in triplicate and the recovery percentage was determined.

### Specificity

Spectral purities of chloroquine and primaquine chromatographic peaks were evaluated using the UV spectra recorded by the DAD to evaluate possible interfering peaks. This analysis was performed employing both standard and sample solutions, at test concentration (0.15 mg/mL of chloroquine and 0.10 mg/mL of primaquine).

### Robustness

Chloroquine and primaquine sample solutions (*n* = 4) were prepared and analysed under the established conditions and by variation of the following analytical parameters: acetonitrile concentration in mobile phase (±2%), mobile phase pH (±0.3) and column temperature (±2°C). Chloroquine and primaquine contents were determined for each condition and the obtained data were submitted to statistical analysis (ANOVA test). Statistical significance was set at *P* <0.05.

### Detection and quantitation limits

Detection and quantitation limits of both methods were calculated based on the ratio of standard deviation of response and slope of calibration curves. For detection limit, a relation of 3.3 was considered, whereas for quantitation limit the relation was 10.

### Analysis of chloroquine and primaquine tablets by UPLC and HPLC methods

Tablet formulations (Farmanguinhos, RJ, Brazil), containing 150 mg chloroquine base and 15 mg primaquine base were assayed using both validated UPLC and HPLC methods. Before the analysis, 20 chloroquine tablets and 20 primaquine tablets were weighed and finely powdered. An accurately weighed portion of the powders, equivalent to about 12.50 mg chloroquine base and 10.00 mg of primaquine were employed to prepare solutions, as described in “Chloroquine and primaquine sample solution”. Analyses were performed using six replicates, in two consecutive days. The contents of chloroquine and primaquine in tablets (% of labeled amount) and coefficient of variation (CV%) were determined for each method. The results were statistically compared using Student’s *t*-test. Statistical significance was set at *P* <0.05. In addition, UPLC and HPLC methods were also compared regarding analysis time, tailing factor and solvent consumption.

## Results

### Method development

**Figure 2:**
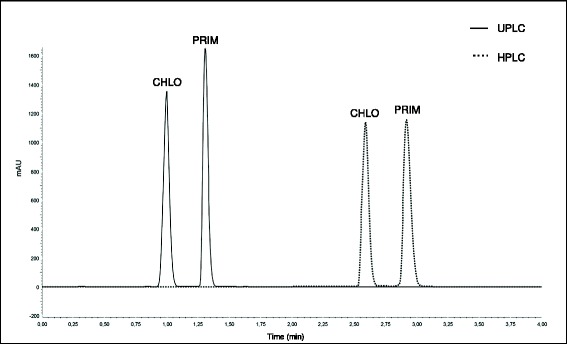
**Chromatograms obtained by UPLC (full line) and HPLC (dotted line) for analysis of chloroquine (CHLO) and primaquine (PRIM) in tablet formulations.**

Chromatographic parameters employed for the UPLC method development were initially adapted from HPLC method already used for routine analysis in our laboratory. The chromatographic parameters were firstly evaluated using C_18_ column and isocratic elution with a mobile phase composed of 60% organic solvent. Under these conditions, the retention factor obtained for chloroquine was considerably low. However, the decrease of organic solvent percentage in mobile phase increased the retention of primaquine peak, leading to a long run time, besides causing peak tailing. The use of gradient elution promoted adequate retention times and peak shapes for both anti-malarial drugs. Acetonitrile instead of methanol showed to be a better choice to assure the adequate separation of peaks. Finally, different pH values of mobile phase were evaluated and better peak symmetries were obtained at pH 3.0. Under these optimized conditions, chloroquine and primaquine peaks eluted at 1.0 and 1.3 min, respectively (Figure 2).

### Validation

#### Linearity

**Figure 3:**
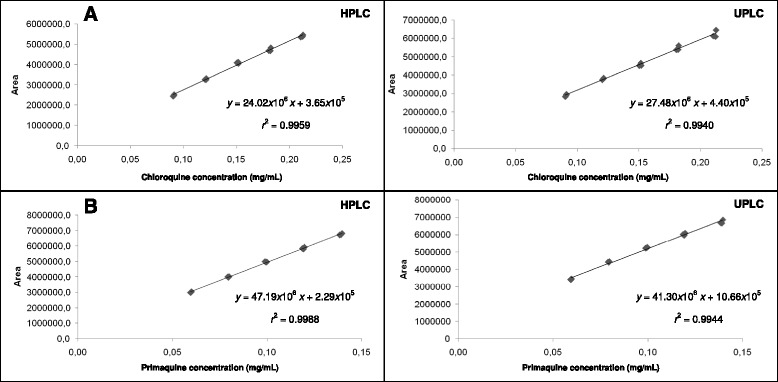
**Calibration curves of (A) chloroquine and (B) primaquine obtained by HPLC and UPLC methods.**

Linear correlation was found between the peak areas and the concentrations of chloroquine and primaquine, in the assayed range, for both HPLC and UPLC methods, using the least squares method. A random pattern of the regression residues was found and no significant deviation of linearity was detected in the assayed range. The regression analysis curves are showed in Figure 3. The regression coefficient (*r*^*2*^) values obtained, higher to 0.99 to both compounds, attested the linearity of the methods.

### Precision and accuracy

RSD and recovery values obtained for chloroquine and primaquine by HPLC and UPLC methods are demonstrated in Table 1. The obtained CV% values, lower than 2.0% [17], assure the HPLC and UPLC method precision. In addition, the employed standard addition experiment demonstrated that both methods were accurate for chloroquine and primaquine analysis, since recovery rates were within the predetermined range of 98.0-102.2% [18].

**Table 1:** **Precision and accuracy data for chloroquine and primaquine obtained by UPLC and HPLC methods**

**Validation parameters**	**UPLC**		**HPLC**	
	**Chloroquine**	**Primaquine**	**Chloroquine**	**Primaquine**
Precision (CV%)				
Intra-day	1.10	1.04	1.10	0.31
Inter-day	1.49	1.05	1.59	1.21
Accuracy (recovery %)	99.22	99.67	99.83	98.11

### Specificity

No interfering peak was found in chromatograms during the optimization step and validation process. Peak purities higher than 99.0% were obtained using DAD for chloroquine and primaquine, since the UV spectra of the peaks obtained with sample solutions were superimposable to the spectra of the peaks obtained with reference standards in HPLC and UPLC methods. It demonstrated that other compounds did not co-elute with the main peaks, attesting the specificity of both methods.

### Robustness

Robustness results obtained for chloroquine and primaquine after variation of analytical parameters are demonstrated in Tables 2 and 3 for HPLC and UPLC methods, respectively. Statistical analysis showed no significant difference between results obtained employing the analytical conditions established for the method and those obtained in the experiments in which variations of some parameters were introduced for chloroquine and primaquine. Thus, HPLC and UPLC methods showed to be robust regarding chloroquine and primaquine contents for changes in acetonitrile concentration in mobile phase during gradient elution (±2%), mobile phase pH in the range of 2.7 to 3.3 and column temperature from 23°C to 27°C.

**Table 2:** **Robustness results for chloroquine and primaquine obtained by HPLC method**

**Conditions**	**Chloroquine**			**Primaquine**		
	**Mean content (%)**	**R.S.D. (%)**	**Retention time (min)**	**Mean content (%)**	**R.S.D. (%)**	**Retention time (min)**
Nominal*	99.59	0.90	2.58	99.77	0.11	2.92
Acetonitile concentration 8-38%	99.92	0.77	2.60	99.82	0.56	3.10
Acetonitile concentration 12-42%	99.49	1.10	2.56	99.68	0.60	2.77
Mobile phase pH 2.7	99.80	1.46	2.58	100.22	1.28	2.87
Mobile phase pH 3.3	100.06	1.65	2.58	100.02	1.31	2.97
Temperature 23°C	99.31	0.67	2.60	99.84	0.17	2.93
Temperature 27°C	99.63	1.13	2.59	99.87	0.26	2.92

*Acetonitrile concentration in gradient 10-40%, mobile phase pH 3.0, temperature 25°C.

**Table 3:** **Robustness results for chloroquine and primaquine obtained by UPLC method**

**Conditions**	**Chloroquine**			**Primaquine**		
	**Mean content (%)**	**R.S.D. (%)**	**Retention time (min)**	**Mean content (%)**	**R.S.D. (%)**	**Retention time (min)**
Nominal*	99.65	0.77	1.00	99.87	0.36	1.31
Acetonitile concentration 8-38%	98.72	1.22	1.08	98.05	1.00	1.45
Acetonitile concentration 12-42%	98.36	1.04	0.73	98.86	1.15	1.27
Mobile phase pH 2.7	99.02	0.92	1.01	98.69	0.50	1.28
Mobile phase pH 3.3	100.53	1.15	1.02	99.12	0.88	1.34
Temperature 23°C	99.97	1.17	1.01	99.11	1.07	1.33
Temperature 27°C	100.42	0.99	0.99	99.26	0.74	1.31

*Acetonitrile concentration in gradient 10-40%, mobile phase pH 3.0, temperature 25°C.

### Detection and quantitation limits

Using the described HPLC method, detection limits were 0.01 and 0.003 mg/mL for chloroquine and primaquine, respectively. Quantitation limits of 0.03 and 0.010 mg/mL were found for chloroquine and primaquine. Using the developed UPLC method, same values were found for chloroquine: 0.01 and 0.03 mg/mL for detection and quantitation limits, respectively. For primaquine, detection limit of UPLC method was 0.007 mg/mL and quantitation limit was 0.022 mg/mL. The low values of detection and quantitation limits demonstrated adequate sensitivity of both methods.

### Comparison of UPLC and HPLC methods for analysis of chloroquine and primaquine in tablets

Samples of chloroquine and primaquine tablet formulations were analysed using both HPLC and UPLC validated methods. The results obtained are showed in Table 4. All analysed samples presented chloroquine and primaquine contents very close to the labeled amount. Student’s *t*-test was employed to compare the mean contents obtained by HPLC and UPLC methods. No significant differences were observed between the results, demonstrating that both chromatographic methods were equivalents regarding chloroquine and primaquine contents. Retention time repeatability and tailing factor of chromatographic peaks were similar for both techniques. In addition, UPLC and HPLC methods complied with all required validation parameters and presented resolution higher than 1.5 between chloroquine and primaquine peaks [19]. However, UPLC method demonstrated lower retention times for the analyte peaks, as showed in Figure 2, allowing high throughput analysis. Total run time of UPLC method (1.5 min) was half of that obtained by conventional HPLC method (3.0 min). In spite of a longer time for column re-equilibration being needed for UPLC method, the total run time was faster, and mobile phase flow rate of UPLC (0.6 mL/min) was lower than HPLC method (1.0 mL/min). These factors promoted a considerably low solvent consumption, reducing the cost of analysis and demonstrating a greener chromatographic approach for routine analysis.

**Table 4:** **Mean contents of chloroquine and primaquine bases in tablet formulations obtained by UPLC and HPLC methods (** ***n*** **= 12)**

**Results**	**UPLC**		**HPLC**	
	**Chloroquine**	**Primaquine**	**Chloroquine**	**Primaquine**
Content (%)	99.15	99.22	99.99	98.79
Content (mg/tablet)	148.73	14.88	149.99	14.82
Standard deviation	1.48	1.04	1.59	1.11
CV (%)	1.49	1.05	1.59	1.12

The developed method offers the advantage over those previously reported using conventional liquid chromatography [13-15] showing a faster chromatographic total run time and better chromatographic performance. Since a high number of samples are needed for quality control analysis, the very fast UPLC separation combined with an adequate efficiency described in this work allows the application of the method for routine analysis.

In addition, the development of simple and reliable methods is essential for qualitative and quantitative determination of anti-malarial drugs, since the problem of counterfeit or substandard anti-malarials is well established all over the world. The adequate quality control of the pharmaceutical preparations marketed nowadays by means of rapid methods is an important tool to assure the treatment efficacy with anti-malarial drugs.

## Conclusions

This study was the first report of simultaneous quantitation of chloroquine and primaquine by UPLC-DAD method. The developed method was shown to be a suitable technique to quantify these anti-malarials in pharmaceutical preparations and may be successfully employed for quality control analysis. Compared to a conventional liquid chromatography method, UPLC promoted lower retention times and better chromatographic performance, leading to shortening of analysis time and great savings in solvent consumption. Chloroquine and primaquine tablets analysed by the validated method showed adequate quality and drug contents in agreement with the labeled amounts.
